# Supplementary material for: Co-Culture of Mesenchymal Stem Cells and Ligamentocytes on Triphasic Embroidered Poly(L-lactide-co-ε-caprolactone) and Polylactic Acid Scaffolds for Anterior Cruciate Ligament Enthesis Tissue Engineering
Source: Int J Mol Sci. 2023 Apr 4;24(7):6714. doi: 10.3390/ijms24076714 (PMC10095212; doi:10.3390/ijms24076714)
Supplement: Supplementary file 1 [file ijms-24-06714-s001.zip › ijms-2280180-supplementary.pdf]

**Supplementary Materials:**

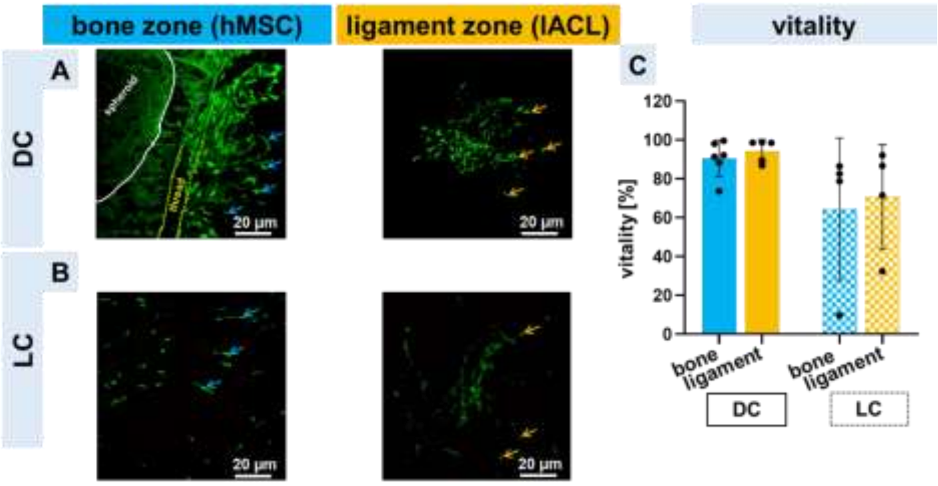


*Supplemental Figure S1 Viable cells on the scaffold surface. Single hMSCs (blue arrows) and single lACL fibroblasts (orange arrows) were emigrating out of the spheroid (borders were marked with the white line) on the polymer thread (the borders of the thread were marked with the yellow line). Scale bars of 20 µm. The calculation of the cell vitality (C) on the bone and the ligament scaffold parts for the direct culture (DC) and the long-time (LC) was based on the percentage cell vitality.*

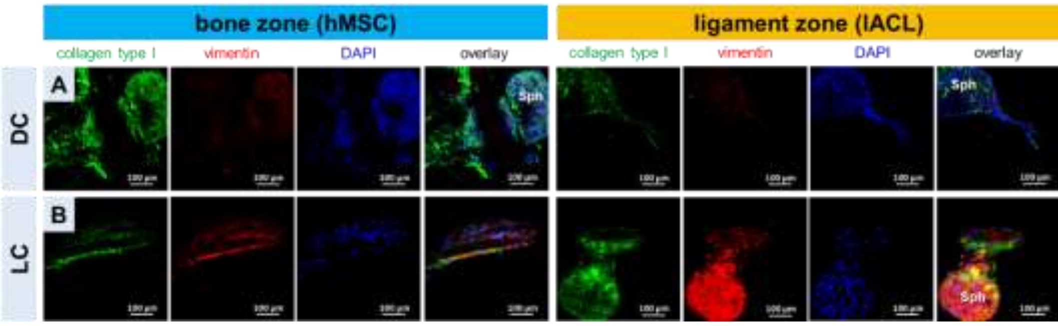


*Supplemental Figure S2* ***Extracellular matrix formation and vimentin expression after 14 days of scaffold culture****. Immunocytochemical staining of collagen type I, mesenchymal marker vimentin and counterstaining of cell nuclei (4′, 6-Diamidino-2-Phenylindole, DAPI) of human mesenchymal stem cells (hMSC) and lapine anterior cruciate ligamentocytes (lACL) spheroids (Sph) which were directly placed on the scaffold zones immediately after the 2 days of spheroid assembly (A, DC) or with spheroids pre-cultured for 14 days (B, long-time: LC) before seeded on the scaffold bone or ligament zones and 14 days of scaffold cultivation. Scale bars of 100 µm.*

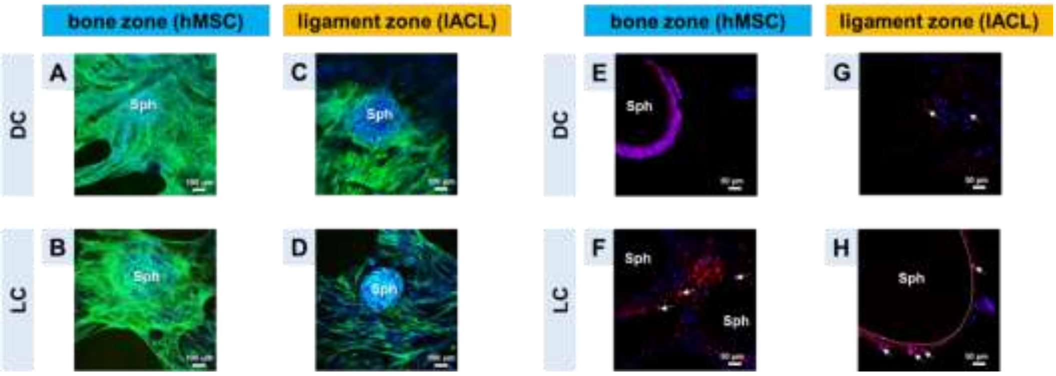


*Supplemental Figure S3 Immuncytochemical staining of F-Actin (green) and Ki-67 (red) and counterstaining of cell nuclei (4′, 6-Diamidino-2-Phenylindole, DAPI, blue) of human mesenchymal stromal cells (hMSCs) and lapine anterior cruciate ligamentocytes (lACL) spheroids (Sph) which were directly placed on the scaffold zones immediately after the 2 days of spheroid assembly (A, C, E, G; DC) or with spheroids pre-cultured for 14 days (B, D, F, H; long-time: LC) before seeded on the scaffold bone or ligament zones and 14 days of scaffold cultivation. Scale bars of 100 µm (A-D) and 50 µm (E-H). The white arrows should highlight the proliferatinge cells. The spheroid border is marked by the white dotted line.*
